# The mechanism of CNP-induced negative inotropic and positive lusitropic responses are dependent on SERCA activity in failing rat ventricle

**DOI:** 10.1186/1471-2210-11-S1-P49

**Published:** 2011-08-01

**Authors:** Lise Román Moltzau, Jan Magnus Aronsen, Silja Meier, Cam Nguyen, Geir Christensen, Ivar Sjaastad, Tor Skomedal, Jan-Bjørn Osnes, Finn Olav Levy, Eirik Qvigstad

**Affiliations:** 1 Department of Pharmacology, Faculty of Medicine, University of Oslo and Oslo University Hospital, Oslo, Norway; 2 Center for Heart Failure Research, University of Oslo, Oslo, Norway; 3 Institute for Experimental Medical Research, Faculty of Medicine, University of Oslo and Oslo University Hospital, Oslo, Norway; 4 Bjørknes College, Oslo, Norway; 5 Department of Cardiology, Oslo University Hospital, Oslo, Norway

## Background

Natriuretic peptides increase in heart failure. C-type natriuretic peptide (CNP) generates cyclic 3’,5’ guanosine monophosphate (cGMP) by activating the NPR-B receptor in cardiomyocytes. There are studies showing CNP-induced negative inotropic and positive lusitropic responses in normal hearts, but less is known about the effects of CNP in failing hearts.

## Results

We investigated the functional effects of CNP in heart failure and suggested that increased activation of SERCA is the main mechanism of CNP-induced negative inotropic and positive lusitropic responses. Increased SERCA activity could cause a faster Ca^2+^ removal from cytosol and thus less Ca^2+^ available for the myofilaments during contraction. Cyclic GMP levels, contraction and relaxation, Ca^2+^ transients, troponin I (TnI) and phospholamban (PLB) phosphorylation were measured in left ventricular muscle strips or cardiomyocytes from Wistar rats with heart failure 6 weeks after myocardial infarction. CNP increased cGMP levels and evoked negative inotropic and positive lusitropic responses concentration-dependently. TnI and PLB phosphorylation also increased in the presence of CNP. The functional responses to CNP were reduced in the presence of a PKG-blocker/cGMP-analogue (Rp-8-Br-Pet-cGMPs), demonstrating activation of the PKG pathway. In the presence of CNP, Ca^2+^ transient amplitude and Ca^2+^ extrusion rate were increased. CNP elicited both negative inotropic and positive lusitropic responses in the presence of the L-type Ca^2+^ channel activator BAY K 8644, whereas in the presence of full activation of the cAMP system by isoproterenol these responses were not seen. This indicates that the downstream targets causing functional responses to CNP were already activated in the presence of isoproterenol. All these results could be explained by an increased SERCA activity and a reduced myofibrillar sensitivity to Ca^2+^ in the presence of CNP, due to increased phosphorylation of PLB and TnI, respectively. An obligatory role of SERCA activation was revealed in a mouse model with cardiomyocyte-specific excision of the SR Ca^2+^-ATPase gene (SERCA-KO). The functional responses to CNP were abolished in 8-week SERCA-KO mice compared to 4-week SERCA-KO mice still possessing some SERCA activity.

## Conclusion

The functional responses to CNP are mediated through the PKG pathway. Activation of SERCA thus seems to be the major and indispensable mechanism of CNP-induced functional responses in failing hearts.

